# “I have learned that nothing is given for free”: an exploratory qualitative evaluation of a social norms edutainment intervention broadcast on local radio to prevent age-disparate transactional sex in Kigoma, Tanzania

**DOI:** 10.1186/s12889-024-20440-w

**Published:** 2024-10-21

**Authors:** Lottie Howard-Merrill, Marjorie Pichon, Alice Witt, Revocatus Sono, Veronicah Gimunta, Enrica Hofer, Fatina Kiluvia, Mengi Alfred, Emmanuel Yohanna, Ana Maria Buller

**Affiliations:** 1 Department of Global Health and Development, London School of Hygiene and Tropical Medicine, London, UK; 2 Department of Education, Practice and Society, Institute of Education, University College London, London, UK; 3 Amani Girls Organization, Mwanza, Tanzania; 4 Gender-Based Violence Specialist, Dar es Salaam, Tanzania; 5 Sociologist and Gender Expert, Dar es Salaam, Tanzania; 6 Kiota Women’s Health and Development, Dar es Salaam, Tanzania

**Keywords:** Transactional sex, Age-disparate sex, Violence against women and girls, Child protection, Social norms intervention, Mass media intervention, Edutainment, Diffusion, Qualitative evaluation, Tanzania

## Abstract

**Background:**

Promising evidence supports the effectiveness of edutainment interventions in shifting norms to prevent violence against women and girls and other harmful practices, yet further research into mechanisms and pathways of impact is needed to inform intervention development, delivery and scale-up. This exploratory qualitative evaluation examined the feasibility and indications of change in attitudes, beliefs, norms and behaviours following the broadcast of a radio drama aired to prevent age-disparate transactional sex in Kigoma, Tanzania.

**Methods:**

Over seven weeks, six episodes were broadcast on local radio weekly, between November and December 2021 in Kigoma, targeting adolescent girls (aged 13–15 years) and their caregivers. Reflection sessions were conducted twice a week with 70 girls across seven schools, supplemented by after-school Girls’ Club listening sessions for a subgroup of 30 girls. We conducted seven before and after focus group discussions, five with girls (*n* = 50), one with men caregivers (*n* = 9) and one with women caregivers (*n* = 9) and analysed them using thematic and framework analysis approaches.

**Results:**

Overall, we found that while girls exhibited significant engagement with the drama, caregiver participation, particularly among men, was low. Thus, no clear changes were detected in men. We did not find any differences in impact based on listening sessions’ attendance vs. home listening. We detected positive changes among girls and women in four thematic areas after listening to the drama: (1) participant’s increasingly challenged perceptions about what kinds of girls and men take part in age-disparate transactional sex, what can be exchanged, and men’s motivations for engaging; (2) there was a shift from attributing blame for age-disparate transactional sex relationships from girls to men; (3) girl’s reported increased agency and confidence to avoid age-disparate transactional sex relationships; and (4) we found a heightened sense of responsibility and recognition for the role of parents, peers and community members in preventing age-disparate transactional sex.

**Conclusions:**

These findings highlight the need for further implementation research to explore ways to effectively engage men. They also underscore the potential of engaging, evidence-based edutainment interventions in fostering spontaneous critical reflection about complex behaviours such as age-disparate transactional sex, and diffusing key messages among target populations without the use of organised diffusion activities.

**Supplementary Information:**

The online version contains supplementary material available at 10.1186/s12889-024-20440-w.

## Introduction

Age-disparate transactional sex (ADTS) – transactional sex relationships between adolescent girls and men aged at least five years older – is an established child protection and public health concern [1]. ADTS relationships are characterised by intersecting inequities related to age, gender, social status and access to resources, and these inequities have negative consequences for girls’ agency and risk of exploitation [2–5]. In sub-Saharan Africa, ADTS is associated with negative sexual and reproductive health and socio-developmental outcomes, including a high prevalence of sexually transmitted infections such as HIV [6, 7], sexual coercion, unplanned pregnancy, intimate partner violence and school dropout among adolescent girls [2, 4, 8, 9].

### Theory: social norms and strategies to change them

Social norms contribute to the perpetuation of global child and adolescent sexual exploitation and abuse [4, 10, 11], including ADTS in Tanzania [3, 4, 8, 12]. Social norms are informal rules that enforce conformity to behaviours based on mutual social expectations about what others do (*descriptive norms*), and what others should do (*injunctive norms*) [13–15]. Norms occur within *reference groups* - people who individuals’ compare themselves to, and act according to their expectations [16] - and are maintained through (the anticipation of) *sanctions* for non-adherence, and *benefits* for adherence [15]. While not implicitly negative, norms can uphold harmful perceptions and behaviours [17–19]. Harmful behaviours such as ADTS can also be upheld by multiple, sometimes conflicting norms, called *norm bundles* [18]. Unlike norms, which are enforced by reference groups, *attitudes* and *beliefs* are individually held and can mirror or diverge from norms [15, 20]. *Pluralistic ignorance* occurs when individuals adhere to norms that contradict their personal values [15, 20], and oftens lead to risky behaviours, including ADTS.

Different strategies have been proposed to change harmful norms. Bicchieri (2016) suggests introducing positive new norms that challenge pluralistic ignorance, for example by providing role models of people engaging in healthy norms that align with individual’s personal values and beliefs [20]. Another suggestion is that norms change occurs upon reaching a critical mass, or tipping point, of individual-level changes in attitudes and/or behaviours that reject the existing norm(s) [15, 21, 22]. Mass communication methods can be an efficient way to provide role models and generate this critical mass by exposing many people to an intervention at once [23]. This can also be aided by *diffusion*, defined as “the process by which (1) an *innovation* (2) is *communicated* through certain *channels* (3) over *time* (4) among the members of a *social system*” [24]. In the case of ADTS, a beneficial “innovation” could be creating an enabling, supportive environment for girls and men to avoid ADTS. Public mediums of communicating new messages (such as radio) are theorised to be more effective at promoting diffusion and changing norms than private mediums (such as individual leaflets and texts), as they facilitate *common knowledge* [25]. The theory posits that those who consume mass media not only adopt new beliefs and attitudes themselves, but also change their beliefs about what others believe (i.e. injunctive norms) as they know that others in their community are also listening [26].

Thus, there is a growing interest in the potential of edutainment to shift norms, based on the premise that media interventions can increase knowledge, foster critical reflection, and shift individual-level attitudes, social norms and behaviours over time [27–30]. Edutainment is defined as “the process of purposely designing and implementing a media message to both entertain and educate in order to increase audience members’ knowledge about an educational issue, create favorable attitudes, shift social norms and change overt behavior” [30]. Edutainment interventions rely on effective messaging strategies, and audiences relating to and engaging emotionally with storylines and characters to achieve their intended changes, a process referred to as *narrative transportation* [31, 32]. The theory proposes that when individuals become absorbed by a story and “transported” into it, they are better able to internalise new perspectives and ideas [33]. Engaged audiences might vicariously experience sanctions and benefits associated with specific behaviours, leading to normative shifts and behavioural change [27]. Audiences are also less likely to resist new ideas if they are distracted by the story, because they are less aware of the edutainment’s persuasive intent [33]. Thus, audience members with high levels of narrative transportation are believed to be more likely to change their attitudes, intentions and behaviours in line with story messages [33, 34].

### Edutainment to prevent ADTS

In the field of violence against women and girls, edutainment interventions have been shown to improve awareness [35], shift attitudes against its acceptability [36], increase support-seeking behaviours and promote supportive behaviours from community members [37, 38]. While evidence of the effectiveness of edutainment to prevent ADTS is limited [39], we can draw on learnings from a 2008–2011 radio campaign targeting ADTS in Tanzania called *Fataki*. In Kiswahili *fataki* refers to explosives or fireworks but is also widely used as a term for an older man engaging in ADTS. An evaluation of the campaign found that it increased discussions about cross-generational sex and bystander intervention in these relationships, and decreased ADTS relationships reported by women; but no impact was found on men’s reports, nor on the norms driving ADTS [40]. A subsequent, qualitative study revealed that although men internalised the negative messages about cross-generational sex from the campaign, they did not identify with the *fataki* character, who was portrayed as a wealthy, promiscuous, older man and described as “deceptive” and “greedy” [41], providing a possible explanation for the lack of behaviour change reported by men.

Building on this evidence, between 2017 and 2020 the Learning Initiative on Norms, Exploitation and Abuse (LINEA)^1^ conducted formative research on the social norms driving ADTS [42], and developed a social norms intervention to prevent ADTS in Tanzania. During formative research we found that the key norms influencing men’s engagement in ADTS were the expectations of men’s heightened sexuality and prowess, and of providing financially for their sexual partners [12, 42]. The key norms driving adolescent girl and young women’s engagement were the expectation of receiving money, gifts or other benefits from their sexual partners, of gaining social status through these gifts and of reciprocating with sex [4, 8, 42]. To address these harmful norms, we developed an intervention that includes two curricula and a radio drama that are designed to take a non-stigmatising, gender-transformative approach. The two curricula target girls and men, respectively, and work at the individual level through increasing their knowledge, skills and motivation to avoid and prevent ADTS, while the radio drama works at the community level and aims to shift social norms associated with ADTS [43].

The radio drama *Msichana wa Kati*, which translates as “The Girl in the Middle,” was developed iteratively over multiple phases [43]. It draws on the principles of edutainment and uses role modelling to allow the audience to vicariously experience the consequences of positive and negative behaviours [31, 44], helping listeners reflect critically on the drivers and consequences of ADTS and how people can help prevent ADTS in their own communities. In particular, the drama aims to promote the protective norms of adults treating all girls in the community “as their own daughter” to protect them against ADTS, and of community members acknowledging they all have a role to play in preventing ADTS. It is made up of 39 episodes, 15–20 minutes each, split across three 13-episode acts. Radio was chosen as the mode of delivery as many Tanzanians have access to this medium, either through traditional radios – 43% of Tanzanians in 2018 [45] – or mobile phones; and because of the potential of radio to target hard-to-reach populations and to be easily and cost-effectively scaled up. The intervention was developed in a collaboration between the London School of Hygiene and Tropical Medicine (LSHTM), and Tanzanian organisations Amani Girls Organization (AGO) and Media for Development International (MFDI), and tested for feasibility and acceptability in Mwanza [46].

This paper reports findings from an exploratory, qualitative pilot evaluation of a condensed delivery of the radio drama component only (not the curricula), implemented in Ujiji Municipal District of Kigoma region, Tanzania over seven weeks in late 2021. This study complements another exploratory, mixed-methods pilot evaluation of a USB flash drive delivery of the radio drama in Kishapu district, Shinyanga region, conducted during the same period [47, 48]. The two studies aimed to assess indications of change following exposure to the *Msichana wa Kati* radio drama, and compare delivery modalities between the two regions of Tanzania. The current study in Ujiji Municipal District had three objectives: (1) compare findings between adolescent girls vis-a-vis their caregivers to better understand differences in intervention engagement and impact between audiences; (2) investigate how the modality of intervention delivery (radio broadcast only versus afterschool listening sessions for adolescent girls) might influence outcomes; and (3) explore shifts in ADTS relevant attitudes, beliefs, social norms and behaviours following exposure to the radio drama. In doing so, we contribute novel insights to improve the development and delivery of edutainment interventions to shift social norms and prevent violence against women and girls.

## Materials and methods

### Study setting

Intervention implementation took place in Kigoma Ujiji Municipal District of Northwestern Tanzania. Kigoma, a rural region situated on the northeast shore of Lake Tanganyika, has a population of approximately 2.5 million people [49], and is one of the nation’s poorest regions, with an estimated poverty rate of 49% compared to the national average of 26% [50]. Notably, Kigoma has a young, culturally diverse population, with 55% aged under 17 years [49], and a large number of long-residing migrants from the bordering countries of Burundi, the Democratic Republic of Congo and Rwanda [51]. The region is among those with the highest rates of gender-based violence in the country, with 34% of ever married women aged 15–49 years experiencing physical, sexual or emotional violence by an intimate partner within the past year, of which 26% was sexual violence [52]. The 2022 Tanzanian Demographic Health Survey also revealed that 17% of girls aged 15–19 years in Kigoma had experienced pregnancy [52].

In addition to this gender-based violence profile, several pragmatic factors influenced the selection of Kigoma region as our study site. Firstly, UNFPA’s longstanding partnership with the organisation Kiota Women’s Health and Development (KIWOHEDE) facilitated the delivery of intervention activities. KIWOHEDE, a Tanzanian non-governmental organisation, specialises in working with marginalised communities, particularly adolescent girls, to address issues such as child sexual abuse, violence, exploitation, adolescent pregnancy, early/forced marriage and school dropout. Secondly, the reach of Joy FM – the community radio station that broadcasted *Msichana wa Kati* – is limited to Kigoma. This ensured that there was no contamination of other regions, particularly Mwanza, where an upcoming cluster randomised-controlled trial of the full LINEA intervention was planned.

### Sampling

We commenced sampling seven schools from Ujiji Municipal District selected purposively with input from the local Government authority Departments of Education, Social Welfare and Community Development based on: (1) Proximity to densely populated areas that could foster ADTS, including motorcycle taxi stands, bus stops and markets; and (2) Schools with high incidences of adolescent pregnancy, truancy and dropouts. KIWOHEDE then recruited adolescent girls aged 13–15 years who attended afterschool Girls’ Clubs in the selected schools, and their caregivers.

### Implementation

For this study, episodes of *Msichana wa Kati* were broadcast on Joy FM six times per week during a 7-week period, between September and December 2021. This included an omnibus on Wednesdays and Saturdays that repeated the previous three episodes. Study participants, in-school adolescent girls and men and women caregivers of in-school girls, were instructed to listen to the drama on Joy FM at home. Three groups of girls (*n* = 30) were also invited to listening sessions during afterschool Girls’ Clubs to test the impact of listener groups versus listening at home only, given adults were likely to control use of household radios. KIWOHEDE staff also held twice-weekly reflection sessions with all girl participants during afterschool Girls’ Clubs, totalling 70 girls across seven schools. Facilitators used guides that included storyline refreshers and discussion prompts to lead group reflections on key themes, storylines and messages from the drama.

### Data collection

Data were collected using focus group discussions (FGDs) at baseline (September 2021) and endline (December 2021), immediately after termination of implementation. Five of the intervention schools were randomly selected for data collection with girls, and two with men and women caregivers, respectively. This ensured that we did not conduct FGDs with members of the same household, which was important for protecting participant’s privacy. Each girl’s FGD included ten participants, while those with adults had nine (*n* = 68) (Table 1). Girl FGD participants were members of the same Girls’ Club and had participated in the intervention together, and three of these groups had attended in-school listening sessions. In caregiver FGDs, the participants’ daughters were members of the same Girls’ Club and had participated in the intervention together. All FGDs took place at a convenient time and in a private and safe location for participants; girls discussions took place in school conference rooms and adults discussions in ward executive’s offices. All FGDs lasted 90–120 min and were audio recorded.

**Table 1:** Characteristics of focus group discussions (FGDs) (*n* = 68)

FGD number	Participant demographic	Number of participants	Where assigned to listen to drama
1	Adolescent girls	10	Home only
2	Adolescent girls	10	Home and school
3	Adolescent girls	10	Home and school
4	Adolescent girls	10	Home and school
5	Adolescent girls	10	Home only
6	Man caregivers	9	Home only
7	Women caregivers	9	Home only

Following a ten-day training delivered by AGO to KIWOHEDE, two KIWOHEDE researchers were selected to facilitate each FGD. They used topic guides that were developed for this study, and tested and refined during the training, which covered: (1) knowledge of ADTS, (2) perceptions of girls and men engaging in ADTS, and (3) preventing ADTS. Baseline FGDs also explored gender norms, and endline FGDs also covered participants’ perceptions and experiences of the intervention (see supplementary files 1, 2, 3 and 4).

### Data analysis

KIWOHEDE staff transcribed the FGD recordings verbatim and translated them from Kiswahili to English. We developed a preliminary thematic coding framework deductively, drawing on the topic guides and the coding framework developed by Pichon et al. (2022) in the companion study [47]. AW then coded four transcripts: one from a baseline and one from an endline FGD with girls, as well as a baseline FGD with men and an endline FGD with women. The research team then finalised the coding framework, refining existing codes and adding inductive codes. AW and MP then double coded all 14 transcripts.

Using a framework approach for the analysis, AW also summarised the data pertaining to each code within each transcript, and input this information into a matrix where columns corresponded to codes and rows corresponded to each FGD [53]. Where participants gave similar responses, these were aggregated together in the summary rather than detailed individually. Changes at the aggregated group level were observed through comparing the summarised baseline and endline findings and were recorded in their own row in the matrix [54]. In August 2022, we held an interpretation meeting with AGO and KIWOHEDE to discuss the preliminary findings. The teams shared contextual details about implementation, data collection, and from their work on preventing violence against girls in the region, which informed and provided further nuance to our findings.

### Ethical considerations

Ethical considerations were paramount throughout this study given the sensitivity of ADTS and the inclusion of 13-15-year-old girl participants. All participants gave written informed consent. All adolescent participants provided written informed assent to participate, and written informed consent was also obtained from their parent or legal guardian. Study staff adhered to LSHTM, AGO and KIWOHEDE’s safeguarding policies and procedures for conducting research with children and vulnerable populations at all times. No safeguarding concerns arose during this study.

The study received ethical approval from Tanzania’s National Institute for Medical Research (NIMR) (Ref: NIMR/HQ/R.8a/Vol.IX/3698) and the LSHTM Ethics Board in the UK (Ref: 22863-1). NIMR approved a Data Transfer Agreement, which allowed us to transfer anonymised data to researchers at LSHTM. In line with General Data Protection Regulation guidance, we adhered to strict procedures to maintain confidentiality and anonymity, such as using anonymised codes to identify participants, storing audio recordings on a password-protected and encrypted platform, and removing all personal identifying information from transcripts.

## Results

While all girls were aged 13–15 years, women caregiver’s ages ranged from 38 to 63 years, and men caregivers were between 34 and 65 years old. All participants enrolled at baseline were retained until endline. In the sections below, we begin by comparing radio listenership and indications of change between adolescent girls vis-à-vis their caregivers (objective 1). We then compare indications of change between adolescent girls who listened to the radio drama both at home and school and those who only listened at home (objective 2), and explore the shifts in attitudes, beliefs, norms and behaviours related to ADTS following exposure to the radio drama (objective 3).

### Radio drama listenership and indications of change by demographic group

Radio drama listenership was highest among girls; most women listened to only a few episodes, mainly at the beginning, and only one man reportedly listened to any episodes. Thus, it is not surprising that we found no indications of change among men, some limited evidence of change among women and strong evidence of change among girls (as demonstrated in the sections below). Among men, we found evidence of gender inequitable attitudes, beliefs and norms at baseline and endline. For example, one man described the association between male sexual prowess and wealth when he stated that for some men who “*have been busy making money*,* and now that he gets it*,* it’s time to brag*”, having many “*lovers*” is a way to prove his wealth (Man caregiver aged 50–60 years, baseline, FGD 6, participant 1). Men also reported gender inequitable beliefs and attitudes related to women and girls, for example when discussing why girls engage in ADTS another man stated: “*sometimes they are influenced by a life of pleasure and money”* (Man caregiver aged 30–40 years, endline, FGD 6, participant 3).

Caregivers described not listening to the drama because they were too busy and/or were not at home during broadcasts. One woman reported that she did not have access to a radio at home, and one man said he preferred to listen to football, which coincided with the broadcasts.

*There is a time you fail to listen to the radio because of being busy. Every time when the radio session starts you find that you are not at home*,* you are busy*,* also lack of radio device [is another challenge to listening] […] In fact, if I had a radio I would try to follow and listen to the radio drama.* (Woman caregiver aged 60–65 years, endline, FGD 7, participant 10)

A few participants also reported that the length and frequency of episodes made it difficult for them to keep up with the story. For example, when asked why he didn’t listen a man responded:

*The duration of the session was not friendly to me […] [and] the episodes were many in succession.* (Man caregiver aged 30–40 years, endline, FGD 6, participant 4).

### Indications of impact of the radio drama by modality of delivery

We found indications of change following intervention exposure among girls and women in four thematic areas: (1) challenging perceptions about ADTS (2) reconsidering who is to blame for ADTS, (3) girls avoiding ADTS, and (4) community members taking responsibility to prevent ADTS. Among adolescent girls, indications of change were evident in both those who listened to the radio drama at home and at school, as well as those who listened only at home, as exemplified throughout the section below in which we highlight where each girl quoted was assigned to listen to the drama. We were not able to detect any differences in indications of change between the two groups.

#### Challenging perceptions about ADTS

Findings suggested that among adolescent girls and women, *Msichana wa Kati* fostered nuanced beliefs and expectations about the nature of exchange in ADTS, as well as the characteristics of people who take part. While all girls reported knowing at baseline that transactional sex relationships occurred between same-aged girls and boys, at endline girls became more aware of adult men’s involvement. The drama also challenged girls’ perceptions or stereotypes about the ‘type’ of man who participates in ADTS. For example, girls learned that educated and professional men with respected roles in community leadership could also engage:

*The radio drama has changed me because before the drama I thought only boys of our age are those who engage in transactional sex with girls*,* but even the adult men who we trust are engaging in transactional sex with young girls. […] I thought only normal [uneducated] men from the community engaged in transactional sex*,* but even those employed in government institutions*,* educated ones also engage in transactional sex.* (Girl aged 13–15 years, endline, FGD 1, participant 2, listened at home only)

Many girls and some women also reported learning from the drama that beyond money and gifts, sex could be exchanged for ‘favours’ like higher marks from a teacher or lifts to school. There was no evidence that men’s knowledge of ADTS shifted in the same way.

*I and my daughters have really enjoyed listening to the radio drama*,* it has led us to learn many things that we had no idea about. We have learnt that transactional sex comes in different forms like free marks by teachers in schools [and] being given a lift by motorcycle riders*,* which ends up harming girls.* (Woman caregiver aged 30–40 years, endline, FGD 7, participant 1)

After listening, girls also better understood men’s possible motivations for gift giving; some girls had previously believed that men built relationships with and offered gifts to girls solely because they wanted to help them, and not because they wanted sex in return:

*It [the radio drama] has changed me because at first*,* I could see a fataki [older man who gives gifts] having friend relationships with young girls*,* but my thought was that he is helping them [without expectation of sexual reciprocation].* (Girl aged 13–15 years, endline, FGD 2, participant 5, listened at home and school)

At endline, girls also described the men who engage in ADTS as using adolescent girls to fulfil their sexual desires before “leaving” or “abandoning” them. One girl shared: “*After the drama I have realised that fataki [older men who give gifts] are there not to help you*,* but rather they are there just to use [you sexually] and leave you”* (Girl aged 13–15 years, endline, FGD 2, participant 5, listened at home and school). Thus, some adolescent girls were able to make more informed decisions about declining gifts from men after the intervention:

*Listening to the radio drama has changed my thoughts a lot. For instance*,* before the drama I only knew that a man can give you gifts without requiring sex*,* so I didn’t think of refusing the gifts. After listening to the drama*,* I have learnt that […] nothing is given for free.* (Girl aged 13–15 years, endline, FGD 4, participant 6, listened at home and school)

#### Attributing blame for ADTS

At baseline, girls believed that community members viewed girls who engage in ADTS negatively: as badly behaved, arrogant and/or poorly raised. Several girls shared sentiments like: “*The community considers a girl who engages in transactional sex to be a prostitute*” (Girl aged 13–15 years, baseline, FGD 1, participant 6, listened at home only). Similarly, at baseline women and men suggested that girls wore provocative clothing to seduce men, and that girls engaged in ADTS out of a desire for luxurious goods and/or lifestyle:

*Sometimes the children themselves become greedy*,* craving the lives of other girls […] But we in the past were not like that. So*,* because of our poor life*,* the child is dissatisfied and begins to enter into the challenge of transactional sex.* (Woman caregiver aged 40–50 years, baseline, FGD 7, participant 4)

As alluded to in this quote, some women suggested that ADTS resulted from a modern hyper-sexualisation of young people brought about by media and globalisation, and stated that “*nowadays our daughters are very passionate about sex*,* young people dream of sex*” (Woman caregiver aged 60–65 years, baseline, FGD 7, participant 6).

After listening to the drama, many adolescent girls adopted more sympathetic attitudes and beliefs towards girls who participate in ADTS. For example, when asked whether she thought the drama changes community perceptions about ADTS one girl stated: “*When I see a girl engaging in transactional sex*,* I cannot directly judge her like ‘This girl is a prostitute’. Instead*,* I have to listen*,* to ask her about the challenges she is going through”* (Girl aged 13–15 years, endline, FGD 4, participant 3, listened at home and school). We observed a similar pattern among women at endline, with more acknowledging that because girls do not have their own money and are very young, they may lack agency to refuse ADTS. For example, at baseline one woman said, “*this [ADTS] happens because of greed*,* they [girls] crave good things, which is why when they find someone to give them they have to take it*”, while at endline the same participant said, “*when a man who provided gifts asks a girl to have sex with him*,* because a young girl has no ability to defend herself then the only solution for her is to provide sex just because of the gifts*” (Woman caregiver aged 30–40 years, endline, FGD 7, participant 1).

We did not identify any indications of change in men’s attitudes towards girls who engage in ADTS. Consistent with this, despite changes in their own views after listening to the radio drama, many girls still felt community members would negatively perceive girls who engaged in ADTS:

*The community considers them [girls who engage in ADTS*,* as] adults. You can find people telling you when they see you walking with them*,* ‘Don’t walk with those girls*,* they are no longer children’. People never ask the girls why they engage in transactional sex with adults.* (Girl aged 13–15 years, endline, FGD 2, participant 2, listened at home and school)

Here we see that although *Msichana wa Kati* seems to have changed girls’ attitudes towards other girls who engage in ADTS, a victim-blaming attitude persisted among the rest of the community.

#### Girls avoiding ADTS

Through the intervention girls learned how to avoid ADTS, and it reinforced their agency to do so. For instance, many girls stated that they learned to confidently refuse men who offer them gifts: “*I am now different compared to the time before I listened to the drama. I am now able to say no to older men or report to parents or teachers when he threatens me”* (Girl aged 13–15 years, endline, FGD 2, participant 2, listened at home and school). Many girls said they learned this during Girls Club reflection sessions, and that they were now able to confidently refuse sexual advances from men and *“say ‘no’ with emphasis”* (Girl aged 13–15 years, endline, FGD 1, participant 7, listened at home only).

One woman also reported learning that girls are able to refuse men’s gifts: *“I have learnt from the drama. There was one girl who rejected the gifts whenever she was given. There are those who agree and there are those who refuse the gifts regardless of their economic situations”* (Woman caregiver aged 40–50 years, endline, FGD 7, participant 3). However, changes in perceptions of girls’ ability to refuse gifts were more common among girls than among women, and we identified no changes of this nature among men, who largely did not listen to the drama.

Importantly, a few girls reported that listening to *Msichana wa Kati* led them to change their behaviours to avoid situations that could lead to ADTS:

*I have learnt a lot [from the drama]. Before*,* I was very fond of asking the motorcycle riders to give me lifts when coming from school to home […] but after I have listened to the drama*,* I have never asked to be given lifts anymore.* (Girl aged 13–15 years, endline, FGD 4, participant 9, listened at home and school)

Moreover, some girls and women stated that girls do have the ability or agency to end ADTS relationships, and after listening to *Msichana wa Kati* emphasised that they would be more likely to do so after receiving advice from friends and family, or seeing the behaviour modelled by others:

*This [ADTS] relationship may reach an end when a girl gets educated from her community on the consequences of transactional sex. Through this*,* a girl can realise that she was doing wrong and therefore decides to stop engaging.* (Girl aged 13–15 years, endline, FGD 1, participant 1, listened at home only)

This finding is in line with the drama’s storylines, in which peers and women in the community advise girls not to get involved in ADTS and/or to end ADTS relationships. This is also in contrast to how this participant responded to the same question at baseline, when she put the responsibility to end the relationship on the girl without mentioning the role of community members, stating that an ADTS relationship would only end if the man started giving her “*little money*” instead of a lot and that “*violated her dignity*”, or she recognized that what “*she is doing is not right*” (Girl aged 13–15 years, baseline, FGD 1, participant 1, listened at home only).

#### Community responsibility for preventing ADTS

We found evidence that after the intervention participants understood the importance of parents, men and the community at large taking responsibility for preventing ADTS. Both before and after the intervention, participants from all groups identified limited parental support as a driver of ADTS. For example, at baseline participants noted how parents not meeting girls’ basic material needs leads to ADTS:

*It [an ADTS relationship] is because of poverty. The parent leaves the child to fend for herself when she is not working. When a girl is in need*,* she is forced to accept transactional sex in order to get soap and oil.* (Girl aged 13–15, baseline, FGD 5, participant 10, listened at home only)

After listening to the drama, girls and women placed greater emphasis on how parents could help prevent ADTS by offering their children guidance and emotional support, and also by not turning a blind eye when girls return home with gifts. For example, when asked at baseline what can be done to prevent ADTS a mother responded, “*the law should look around*,* even the girls should be sent to jail*” (Woman caregiver aged 40–50 years, baseline, FGD 7, participant 5), while at endline the same participant said:

*Sometimes we female parents […] influence [our daughters] to engage in transactional sex. You cannot see your daughter coming home with new clothes or vegetables and then remain silent without asking where they are from. The problem [is that] parents normally enjoy the gifts brought by their daughters without knowing that they are driving them into destruction. So as parents we are required to provide counselling to our children.* (Woman caregiver aged 40–50 years, endline, FGD 7, participant 5)

Participants also suggested that it was important for parents, and particularly mothers, to be emotionally close to their children so that they would feel more comfortable seeking help from them:

*The best way to help them is for parents to create a friendship environment with their children so that whatever challenge a girl faces*,* for example if a man tells her that ‘I love you’*,* she can go and tell her mother about it and get helped. The other thing is to sit down with them and provide them with advice.* (Girl aged 13–15 years, endline, FGD 5, participant 4, listened at home only)

As highlighted by these quotes, following the intervention more girls and women emphasised the importance of family and community members in preventing ADTS. Several girls drew on the character of Mama Prita, a supportive woman in the community who gives girls advice and material support, to outline the role that women can play in preventing ADTS:

*My primary school teacher had the same character as Mama Prita […] There was one girl whose behaviour was not good*,* so when the teacher saw her*,* she called her parent and later sat down with the girl and advised her. In the end*,* the girl changed her behaviour and performed well in her final examination.* (Girl aged 13–15, endline, FGD 1, participant 4, listened at home only)

After listening to the drama, some girls also emphasised that ADTS prevention should not only be the responsibility of women and girls, but also men: “*The entire community has the responsibility of helping girls. The responsibility is not for women only*,* we also need men’s efforts. We need to eradicate [the] poor perception that a mother is the only person responsible for a girl child in the family and not a father”* (Girl aged 13–15 years, endline, FGD 4, participant 6, listened at home and school).

Some girls also described a growing expectation that community members help girls who engage in ADTS, particularly those who become pregnant, which was also a major drama storyline:

*At first*,* I thought that when a girl who is engaging in transactional sex gets pregnant*,* the community will see her as useless. But after I listened to the drama then I came to realise that a girl can have a pregnancy but still be valued by the community.* (Girl aged 13–15 years, endline, FGD 2, participant 9, listened at home and school)

Finally, there was a noted change in girls’ recognition of the role they can play in sharing knowledge gained from *Msichana wa Kati* to prevent ADTS. One girl recounted learning to advise her peers to avoid risky behaviours: “*[The drama] has changed me*,* I have learnt that […] if my friend is seduced then I have to advise her to refuse engaging with the man”* (Girl aged 13–15 years, endline, FGD 4, participant 10, listened at home and school). A few girls also described how, during the intervention, they had tried to intervene to support peers who they thought could be at risk of ADTS:

*I discussed [the radio drama] with my neighbours to educate them*,* since there is a girl in our neighbourhood who engages in risky behaviours [ADTS or behaviours which could lead to it]. So*,* I used the drama and discussion as the reference to counsel her to be careful with men. I told her that not all men who tell you that you are beautiful are good.* (Girl aged 13–15 years, endline, FGD 4, participant 10, listened at home and school)

Girls emphasised that younger girls were more likely than older girls to respond to advice about ending ADTS relationships, perhaps because older girls were more intentional about engaging: “*It is easier for the young girls to change when they are warned*,* especially by adults*,* but for the older girls it becomes difficult to change because [they feel grown up and independent]”* (Girl aged 13–15 years, endline, FGD 1, participant 2, listened at home only). This finding highlights the importance of intervening with young adolescent girls early, before they have started engaging in ADTS, and when they are more receptive to advice.

## Discussion

This exploratory qualitative evaluation has contributed important evidence on how the *Msichana wa Kati* radio drama might shift attitudes, beliefs, social norms and behaviours to prevent ADTS (study objective 3). Our findings fell into four thematic categories: (1) challenging perceptions about ADTS (2) reconsidering who is to blame for ADTS, (3) girls avoiding ADTS, and (4) community members taking responsibility to prevent ADTS. These results are also in line with the positive indications of change found in a mixed-methods exploratory evaluation of a USB flash drive-based delivery of *Msichana wa Kati* in Shinyanga Region. In Shinyanga, however, implementation at the household level had the added impact of sparking conversations between girls and caregivers about ADTS [47]. These indications of change serve LINEA’s over-arching aim to create normative and behavioural change through counteracting previously held normative expectations of silence and inaction from families and communities, and fostering a communally held responsibility for preventing ADTS and supporting adolescent girls’ development.

The diversity of perspectives captured in the findings provide an avenue to consider the acceptability and efficacy of different modalities of delivery for different populations. Girls demonstrated the clearest indications of change, and there was no evidence of variation based on whether girls took part in school-based listening sessions in addition to listening at home (study objective 2). Indications of change were much weaker among women, and not detectable among men (study objective 1), likely because only some women reported listening to the radio drama, and only one man reported listening to any episodes. Among men we found evidence of harmful norms linked to ADTS at both baseline and endline, which were in line with those found in our formative research, such as the expectation of men to have heightened sexual prowess [12, 42], and for girls to gain social status through gifts [4, 8, 12], highlighting how difficult it can be to change deeply engrained norms [20]. It is possible that girls were more engaged in the intervention then their caregivers because the content was more appealing to girls, or because we targeted participants through schools, highlighting the need to directly target adults when advertising the drama, and to broadcast at different times of day when caregivers could listen on their own.

The companion study in Shinyanga found that participants who took part in family discussion groups listened to more episodes, and those who listened more showed greater indications of change. Men’s engagement was also much higher than in this study, likely due to the delivery of the intervention on USB flash drives [47]. In Shinyanga we also found that men’s engagement varied by age, with older men reporting more time to listen than younger men, suggesting that strategies to engage men should also be adapted for different age groups [47]. Women, and particularly men’s low engagement with the intervention in this study may in part also be explained by the radio broadcast occurring at inconvenient times, highlighting the importance of increasing the frequency of broadcasts over different times of day and days of the week, as well as using a variety of modes of delivery, for example broadcasting in community spaces or making the drama freely available online.

Findings from this study suggest that *Msichana wa Kati* led to shifts in participant’s attitudes and beliefs by challenging commonly held (mis-)perceptions about ADTS. Examples include challenging the stereotype that only ‘uneducated’ men participate in ADTS and undermining the tendency to stigmatise ‘cunning and greedy’ girls who engage in ADTS. In this study and our previous exploratory evaluation [47], listening to the drama also appeared to have instilled new, more empathetic attitudes towards girls impacted by ADTS, such as women caregivers acknowledging the need to not ostracise girls who experience ADTS and its negative consequences, including unintended pregnancy, as role modelled by some of the radio drama characters. These might represent two of the important steps outlined by Bicchieri (2016) that are needed to begin to shift harmful social norms [20]: (1) challenging pluralistic ignorance in communities, and (2) creating positive new norms. The use of an edutainment approach appeared to help listeners empathise with others in their community, encouraging them to identify with the characters and immerse themselves in the story through a process of ‘transportation’, which can also support future normative change [31].

Despite the condensed intervention delivery, and there only being three months between baseline and endline data collection, *Msichana wa Kati* also demonstrated the potential to begin to challenge the hard-to-shift norms around sexual reciprocity and gifting, and the victim blaming narratives that drive ADTS. This exploratory evaluation, therefore, supports evidence that interventions to shift social norms driving violence against women and girls can produce tangible results within programmatic timeframes [55]. We also found that while girl participants adopted more favourable attitudes towards girls who partake in ADTS, they believed that these girls would still be stigmatised by other members of their community. This suggests that no common knowledge was created during this intervention, as participants did not know if others in the community were also listening to the radio drama or not [25]. To promote common knowledge, future broadcasts could highlight their wide listenership by sharing messages from diverse listeners on air, which could also help catalyse norm change [25]. A longer intervention period could have also allowed for more conversations about the drama to occur within the community, facilitating this process [24].

Finally, we also found evidence of behaviour change from girls after listening to *Msichana wa Kati.* Girls reported learning to refuse gifts from men, or to stop asking for free rides to school, thereby asserting boundaries and making choices that prioritise their well-being as a route to preventing ADTS, in line with their evolving developmental capacities [56]. There was also evidence that they started supporting their peers to prevent ADTS by talking to at risk girls in their community about it. Adolescent girls reported taking inspiration from radio drama characters and described using storylines as prompts for discussions with their peers to encourage them to avoid ADTS relationships. There is evidence that this occurred within afterschool Girls’ Clubs, but also outside of them, without the use of formal, organised diffusion exercises integrated in the intervention [57]. This is a promising finding as it suggests that key messages from *Msichana wa Kati* have the potential to spontaneously diffuse throughout the community, generating the critical mass needed to create norms change [24].

### Limitations and strengths

While we found evidence of strong indications of change, especially among girls, it is possible that this may have been, at least in part, a reflection of social desirability bias. Moreover, while girls and women reported more desirable attitudes, beliefs and norms linked to ADTS at endline, there is less evidence that these translated into behaviour change. The intervention was implemented over a short period of seven weeks, which may have impacted the degree to which we were able to observe changes in knowledge, attitudes, norms and behaviours, given that such shifts take considerable time. Additionally, due to the small sample and short implementation period, our findings did not provide insight into the point at which the intervention might reach a critical mass of individual-level changes to trigger community-level norm change.

Moreover, our small sample size and lack of a control group make it difficult to attribute with certainty all changes we observed to the intervention. Throughout the results, however, we present quotes in which the participant’s themselves attribute the change they experienced to the radio drama, or we highlight differences in the data between baseline and endline that indicate change could be attributed to the drama. We also only had one FGD for women and men, respectively, which limited the data available from these groups, and may have affected the degree to which our findings represented their views. Importantly, only one man reported listening to any of the radio drama. Men identified the main barriers to listening as being too busy or not at home during broadcasts, highlighting the need for multiple broadcasts at different times of day, and days of week at an appropriate frequency to improve engagement with diverse populations. Our study also had several strengths, including the use of longitudinal data and FGDs, which allowed us to highlight the heterogeneity in the study population and the bundles of sometimes contradictory norms linked to ADTS in intervention communities [18].

## Conclusions

Our findings suggest that this condensed delivery of *Msichana wa Kati* can begin to shift attitudes, beliefs, social norms and behaviours through challenging commonly held misconceptions about ADTS, including that even ‘educated’ and ‘respected’ men in the community can be engaged in these relationships, and that blame for ADTS should not be placed solely on adolescent girls, but on men and the wider community as well. We found considerably more evidence of change among girls, who reported listening to the entire radio drama and attended reflection sessions twice a week, than among adult participants, and particularly men caregivers who were less exposed to and engaged with the intervention.

Engaged listeners appeared to discuss drama key messages with friends without formal encouragement, suggesting that highly entertaining content can fosters spontaneous diffusion among listener’s networks. To meet the needs of varied listener populations, in this case to reach both men and adolescent girls, mass media interventions can be delivered with added frequency, such as multiple daily broadcasts, and using a range of modes of delivery. Our results support initiatives to develop and deliver edutainment interventions on public broadcast, including those targeting adolescent girls, but highlight the importance of additional investigation into how best to encourage engagement with adult caregivers, in particular men.

## Electronic supplementary material

Below is the link to the electronic supplementary material.

Supplementary Material 1

Supplementary Material 2

Supplementary Material 3

Supplementary Material 4

## Data Availability

The datasets used and analysed during the current study are available upon reasonable request. You can request this data for use in ethically approved research via the LSHTM Data Compass repository at 10.17037/DATA.00003750 or by emailing researchdatamanagement@lshtm.ac.uk.

## Footnotes

1 More information about the LINEA initiative is available at our website: https://www.lshtm.ac.uk/research/centres-projects-groups/linea

## Declarations

### Disclaimer

The findings, conclusions and opinions expressed in this manuscript are those of the authors and do not represent the official positions of UNFPA, or any of the other affiliated universities or organisations.
