# Supplementary material for: “I have learned that nothing is given for free”: an exploratory qualitative evaluation of a social norms edutainment intervention broadcast on local radio to prevent age-disparate transactional sex in Kigoma, Tanzania
Source: BMC Public Health. 2024 Oct 21;24:2907. doi: 10.1186/s12889-024-20440-w (PMC11495037; doi:10.1186/s12889-024-20440-w)
Supplement: Supplementary file 1 — Supplementary Material 1 [file 12889_2024_20440_MOESM1_ESM.docx]

**PRE- RADIO DRAMA: ADULT FOCUS GROUP DISCUSSION**

**KIGOMA**

Amani Girls Organization and London School of Hygiene and Tropical Medicine (LSHTM)

Notes to facilitator:

1. Text in **bold** is to be read aloud, to help explain to the participants what is happening.
2. Read aloud numbered questions in normal text.
3. Text in *italics* is to help you manage your time, suggest further questions, and organise the discussion.
4. Use the time guides for each section to help you structure the focus group.

| *10 minutes: Introduction* | |
| --- | --- |
| Hello, my name is [your name] and my colleagues are [their name(s)]. We are here from Kiwohede, a reproductive health, child development and development and advocacy organisation. Thank you for joining us today. We are conducting discussions before and after the release of a new radio drama to test whether it has an impact on the health and wellbeing of girls, their families, and their communities. We are recording today’s discussion so that we can remember everything that you said. We might use your words as examples when writing up our reports, but your name will not appear anywhere.  Our group discussion will last no more than 2 hours. I will be leading the conversation and my colleagues will be helping me ensure everything runs smoothly. Your participation today is voluntary and confidential. This means that we will not share your names or information that would allow someone outside this group to know who was in this group. No information will be publicly shared that would identify anyone in this group.  Before we begin our discussion, I need to ask you if you each agree to keep what is said in the group today to yourself. This means not telling others outside this group what you heard here today after we finish talking. It also means respecting each other’s privacy by not saying to others who was here or who made specific comments. Please also do not mention your name or anyone else’s name during the session as we are recording it. Any person who is not able to do this may leave the group now or at any time. Do you agree?  We hope that each of you will feel free to express your opinions fully and share your own beliefs about the topics that we will be discussing. You are each free not to answer any question or to leave the discussion whenever you like. Your views and experiences are very important to us.  Before we start, I want to establish a few ‘ground rules’, for our discussion. First, remember that there are no right or wrong answers. We will respect all of the points of view shared in this discussion. We will encourage each other to share. The researchers and participants will listen to others talking, and wait until they are finished before sharing their views.  Does anyone have any questions at this stage?  As you see, we have assigned each of you a number. Please say your number each time before speaking, and each time before addressing another participant in the group. If you forget to say it that is okay, you can say it after you have finished speaking. Now let’s play a game to practice.  *Use the ‘throwing a ball’ exercise to get participants to practice using the numbers. Do the exercise in a speedy style – you only have 5 minutes for this.*  *Throwing a ball*  *Step A: Ask the participants to stand and form a circle.*  *Step B: Explain that you are going to throw a ball to somebody and that person should then say their number and respond to the question: “what is your favourite animal?”*  *Step C: Once the first participant has answered, they should say the number of another participant that they are throwing the ball to, who will catch the ball and then say their own number and respond to the same question. (Remind participants that it is OK for them to repeat what the others have said.)*  *Step D: When everyone has received and thrown the ball and said the numbers correctly, take the ball back and begin the group discussion.* | |
| *15 mins: General questions* | *OBJECTIVES* |
| 1. What do you like most about living in your community?   *For each question, collect brief responses from all participants, and then move on.*   1. Do you think people in your community values boys and girls in the same way or in a different way?   *Let them express themselves freely, then probe if necessary:*  Can you give me examples?  What do you think about this?  *For each question, collect two or three responses, and then move on.*   1. What do you think are the main challenges girls face in this community?   *Let them express themselves freely, then probe if necessary:*  How do adolescent girls respond to these challenges?  Do they get any help from community members? Who?  *For each question, collect two or three responses, and then move on.*   1. What do you think are the main challenges boys face in this community?   *Let them express themselves freely, then probe if necessary:*  How do adolescent boys respond to these challenges?  Do they get any help from community members? Who?  *For each question, collect two or three responses, and then move on.* | To open the focus group discussion.  To make participants comfortable with the format of the discussion.  To get to know the participants better.  To explore community perceptions of boys and girls.  To learn about the challenges that these perceptions can cause. |
| *20 mins: Girls and transactional sex* | *OBJECTIVES* |
| Now we are going to talk about something called transactional sex / sugar daddy relationships / fatakis. This is exchange of material support or other benefits for sex between a young girl, and a man 5-10 years older than her. This happens outside of marriage, and it is different from commercial sex work.  *For the following questions, encourage spontaneous conversations to happen between participants if they arise.*   1. Is this something that happens in your community?   *Let the participant express themselves freely. If they answer yes ask:*   - - 1. Does it happen a lot?     2. Does it happen with girls aged 13-15 (or slightly older or younger)?   *If they answer no, then ask:*   - - 1. Have you heard about it happening elsewhere?   *For each question, collect responses from all participants who volunteer answers, and then move on.*   1. Can you tell me about what happens in transactional sex relationships between adult men and young girls?   *Let the participant express themselves freely. Then ask:*   - - 1. How do you think these relationships start?   *Probe: Explore gift giving: is it men’s initiative, do girls ever provide a signal to men?*   - - 1. What makes adult men pursue young girls?   *Probe: Explore gift giving, explore physical characteristics or behaviours of girls.*   1. Once a young girl accepts gifts from an adult man, is she obliged to give him something in return?   *Probe: Explore what does she need to give in return?*   - - 1. Does this change if the girl is very young / older?     2. Does this change if a girl is physically developed, or whether she has not reached puberty yet?   *Probe: Ask about what makes a girl ready for sex, and therefore ready to make decisions about transactional sex.*   - - 1. Does this change if the girl is in a difficult financial situation?     2. Does this change if the man gives her a very small or very large gift?   *For questions 8a, 8b and 8c, explore power dynamics. Is it men’s decision? Is it girls’ decision?*   1. How do these relationships normally come to an end?   *Probe: Explore power dynamics. Is it men’s decision? Is it girls’ decision?* | To familiarize participants with our definition of age-disparate transactional sex.  To invite participants to describe transactional sex relationships they know about.  To learn how common participants believe age-disparate transactional sex is in their community.  To explore community understandings of how age-disparate transactional sex relationships begin, and who tends to start them.  To explore community views about reciprocity in age-disparate transactional sex relationships.  This will relate to ideas about girls’ ability to say no and refuse age-disparate transactional sex.  To explore community understandings of how girls and men leave age-disparate transactional sex relationships. |
| *15 mins: Community perspectives of girls and transactional sex* | *OBJECTIVES* |
| Now we are going to start talking about what people in the community think about transactional sex. We are going to think about their views of both girls and men who take part. Let’s start with girls.   1. What do people in your community think about girls who take part in transactional sex?   *Probe around community judgements if necessary: for example, are they judged for being ‘easy’, or do people think they are clever...?* *Make sure you ask about the following groups:*   1. What do parents in your community think about girls who take part in transactional sex?   *Get responses from two or three participants and then move on.*   1. What do teachers, community leaders, or other professionals think about girls who take part in transactional sex?   *Get responses from two or three participants and then move on.*   1. We have talked about girls who engage in transactional sex, but many girls do not engage in transactional sex. What do people in your community think about girls who do not take part in transactional sex?   *Prove around community judgements if necessary: for example, are they clever…?* | To explore how people in the community value girls who take part in age-disparate transactional sex.  To explore how people in the community value girls who do not take part in age-disparate transactional sex. |
| *20 mins: Men and transactional sex* | *OBJECTIVES* |
| Now we are going to talk about how people in the community think about men who take part in transactional sex.   1. Why do you think older men engage in TS with girls?   *Let the participant express themselves freely. Probe around provision (gift giving, providing for their families etc.) and sexuality (desire to try new things, desire to have multiple partners etc.).*  *Then ask:*   1. What do you think are the benefits for men? 2. What do you think are the risks for men?   *For each question, collect three of four responses, and then move on.*   1. What do people in your community think about men who take part in transactional sex with girls?   *Let them express themselves freely, then probe around community judgements if necessary. For example, do people admire these men? Do they think that these men care about girls?*  *You can also ask:*   1. What do adults in your community think about men who take part in transactional sex? 2. What do teachers, community leaders, or other professionals think about men who take part in transactional sex?   *For each question, collect three of four responses, and then move on.*   1. What do people in your community think about men who do not take part in transactional sex with girls?   *Let them express themselves freely, then probe around community judgements if necessary. You can ask similar questions: do people admire these men? Do they think that these men care about girls?*  *You can also ask:*   1. What do adults in your community think about men who do not take part in transactional sex? 2. What do teachers, community leaders, or other professionals think about men who do not take part in transactional sex?   *For each question, collect three of four responses, and then move on.* | To explore community perceptions about men’s motivations are to engage in age-disparate transactional sex, and the benefits and risks of age-disparate transactional men.  To explore how people in the community value men who take part in age-disparate transactional sex.  To explore how people in the community value men who do **not** take part in age-disparate transactional sex. |
| *10 mins: Responsibility to prevent transactional sex* | *OBJECTIVES* |
| 1. What do you think could be done to support girls to avoid these risks?   *Let them express themselves freely, then if necessary probe around support at school, at home, by the government...?*  *Try to encourage discussion between participants about this. Aim to get a range of views from the participants.*   1. Who in the community can support them, and how?   *If they don’t mention a specific group, ask about peers, parents, teachers...?*  *For each question, collect three of four responses, and then move on.* | To learn how participants believe that age-disparate transactional sex could be prevented.  To end the interview on a positive note. |
| *5 mins: Closing the discussion* | |
| We have come to the end of the discussion. Thank you for helping us understand more about your views. We have talked about many things today. Please remember that we agreed to keep this discussion confidential. Please do not share what was said here with others outside the group.  People will be curious and you may have to say something. I suggest you tell them that we were discussing how to improve a radio drama. Please do not give further details, so that we can maintain confidentiality.  How does that sound to you? Do you have questions for me? If you would like to speak with me further in private, I will be here for a time after we end. If you have questions on another day, you can contact the study supervisor Revocatus Sono.  Thank you for your time. | |
