# Supplementary material for: “I have learned that nothing is given for free”: an exploratory qualitative evaluation of a social norms edutainment intervention broadcast on local radio to prevent age-disparate transactional sex in Kigoma, Tanzania
Source: BMC Public Health. 2024 Oct 21;24:2907. doi: 10.1186/s12889-024-20440-w (PMC11495037; doi:10.1186/s12889-024-20440-w)
Supplement: Supplementary file 3 — Supplementary Material 3 [file 12889_2024_20440_MOESM3_ESM.docx]

**POST- RADIO DRAMA: ADULT FOCUS GROUP DISCUSSION**

**UJIJI**

Amani Girls Organization and London School of Hygiene and Tropical Medicine (LSHTM)

Notes to facilitator:

1. Text in **bold** is to be read aloud, to help explain to the participants what is happening.
2. Read aloud numbered questions in normal text.
3. Text in *italics* is to help you manage your time, suggest further questions, and organise the discussion.
4. Use the time guides for each section to help you structure the focus group.

| *10 minutes: Introduction* |
| --- |
| Hello again. To remind you, my name is [your name] and my colleagues are [their name(s)], and we are from Kiwohede, a reproductive health, child development and development and advocacy organisation. Thank you for joining us today. We are conducting discussions before and after the release of a new radio drama to test whether it has an impact on the health and wellbeing of girls, their families, and their communities.  Like last time, our group discussion will last no more than 2 hours. I will be leading the conversation and my colleagues will be helping me ensure everything runs smoothly. Your participation today is voluntary and confidential. This means that we will not share your names or information that would allow someone outside this group to know who was in this group. No information will be publicly shared that would identify anyone in this group.  Before we begin our discussion, I need to ask you if you each agree to keep what is said in the group today to yourself. This means not telling others outside this group what you heard here today after we finish talking. It also means respecting each other’s privacy by not saying to others who was here or who made specific comments. Please also do not mention your name or anyone else’s name during the session as we are recording it. Any person who is not able to do this may leave the group now or at any time. Do you agree?  We hope that each of you will feel free to express your opinions fully and share your own beliefs about the topics that we will be discussing. You are each free not to answer any question or to leave the discussion whenever you like. Your views and experiences are very important to us.  Does anyone have any questions at this stage?  Before we start, I want to remind you of the ‘ground rules’ from last time, for our discussion today. First, remember that there are no right or wrong answers. We will respect all of the points of view shared in this discussion. We will encourage each other to share. The researchers and participants will listen to others talking, and wait until they are finished before sharing their views. Finally, the participants should not share the personal experiences or views expressed by others once they have left the discussion.  Does anyone have any questions at this stage?  Let’s begin! |
| *30 mins: Radio drama questions* |
| The first questions today are about your experience of listening to the radio drama.   1. Thinking about the entire radio drama, which stories did you like most? Why?   *Ask for examples from three or four participants and then move on.*   1. Again, thinking about the entire radio drama, which stories did you like least? Why?   *Ask for examples from three or four participants and then move on.*   1. How many of the radio drama episodes would you say that you have listened to?   Would you say less than half; half; more than half; or all episodes?  *Get responses from as many participants as possible, and then move on.*  *For people who said half or less than half ask:*   - 1. What was the reason you did not listen to all of them?   *Probe: Were they too busy, not able to access a radio, not that interested...*   1. What do you think about the timing and frequency of the radio drama?   *Probe: Time of the day, frequency of the episodes...*   1. Did anyone speak other people about what they heard in the radio drama between the sessions?    1. Who did you speak to?    2. What did you discuss?   *Get responses from three or four participants and then move on.*   1. Did anyone not speak to other people about what they heard in the radio drama between sessions? 2. Why not?   *Get responses from any participants who volunteer to share.* |
| *20 mins: Transactional sex in the radio drama* |
| We are now going to talk about some of the stories that you heard about in the radio drama.  *NOTE: Before starting this section, remind participants that they should NOT share personal details about themselves or others. They should share the story without sharing information that might make the person identifiable.*   1. Do you know anyone in your community who has had a similar experience to Amali?   *Let them express themselves freely, then probe if necessary.*     - - 1. Can you tell me about what happened?     2. What did you think about the support she received from her friends, family and community?     3. Is this something that happens in your community?   *Get responses from any participants who volunteer to share.*   1. Do you know of anyone in your community who has had a similar experience to Tuma?   *Let them express themselves freely, then probe if necessary.*   - - 1. Can you tell me about what happened?     2. What did you think about the support he received from his friends, family and community?     3. Is this something that happens in your community?   *Get responses from any participants who volunteer to share.*   1. Do you know of anyone in your community who has had a similar experience to Baba Amali (Tizo)?   *Let them express themselves freely, then probe if necessary.*   - - 1. Can you tell me about what happened?     2. Do you think fathers supporting their daughters something that happens in your community?   *Get responses from any participants who volunteer to share.*   1. Do you know of anyone in your community like Mama Amali (Zawadi)?   *Let them express themselves freely, then probe if necessary.*   1. Can you tell me about what happened? 2. Do you think mothers supporting their daughters something that happens in your community?   *Get responses from any participants who volunteer to share.* |
| *20 mins: Girls and transactional sex* |
| Reflecting on what you heard in the radio drama, we are going to talk again about transactional sex in your community. You can use examples (stories or characters) to help you answer the questions.  *For the following questions, encourage spontaneous conversations to happen between participants if they arise.*   1. Is this something that happens in your community?   *Let the participant express themselves freely. If they answer yes ask:*   - - 1. Does it happen a lot?     2. Does it happen with girls aged 13-15 (or slightly older or younger)?   *If they answer no, then ask:*   - - 1. Have you heard about it happening elsewhere?   *For each question, collect responses from all participants who volunteer answers, and then move on.*   1. Can you tell me about what happens in transactional sex relationships between adult men and young girls?   *Let the participant express themselves freely. Then ask:*   - - 1. How do you think these relationships start?   *Probe: Explore gift giving: is it men’s initiative, do girls ever provide a signal to men?*   - - 1. What makes adult men pursue young girls?   *Probe: Explore gift giving, explore physical characteristics or behaviours of girls.*   1. Once a young girl accepts gifts from an adult man, is she obliged to give him something in return?   *Probe: Explore what does she need to give in return?*   - - 1. Does this change if the girl is very young / older?     2. Does this change if a girl is physically developed, or whether she has not reached puberty yet?   *Probe: Ask about what makes a girl ready for sex, and therefore ready to make decisions about transactional sex.*   - - 1. Does this change if the girl is in a difficult financial situation?     2. Does this change if the man gives her a very small or very large gift?   *For questions 8a, 8b and 8c, explore power dynamics. Is it men’s decision? Is it girls’ decision?*   1. How do these relationships normally come to an end?   *Probe: Explore power dynamics. Is it men’s decision? Is it girls’ decision?*   1. Do you think listening to the radio drama has changed at all the way you think about transactional sex? |
| *15 mins: Community perspectives of girls and transactional sex* |
| Now we are going to start talking about what people in the community think about transactional sex. We are going to think about their views of both girls and men who take part. Let’s start with girls. You can use examples (stories or characters) to help you answer the questions.   1. What do people in your community think about girls who take part in transactional sex?   *Probe around community judgements if necessary: for example, are they judged for being ‘easy’, or do people think they are clever...?* *Make sure you ask about the following groups:*   1. What do parents in your community think about girls who take part in transactional sex?   *Get responses from two or three participants and then move on.*   1. What do teachers, community leaders, or other professionals think about girls who take part in transactional sex?   *Get responses from two or three participants and then move on.*   1. We have talked about girls who engage in transactional sex, but many girls do not engage in transactional sex. What do people in your community think about girls who do not take part in transactional sex?   *Prove around community judgements if necessary: for example, are they clever…?*   1. Do you think listening to the radio drama has changed at all the way you think about transactional sex? |
| *20 mins: Men and transactional sex* |
| Now we are going to talk about how people in the community think about men who take part in transactional sex. You can use examples (stories or characters) to help you answer the questions.   1. Why do you think older men engage in TS with girls?   *Let the participant express themselves freely. Probe around provision (gift giving, providing for their families etc.) and sexuality (desire to try new things, desire to have multiple partners etc.).*  *Then ask:*   1. What do you think are the benefits for men? 2. What do you think are the risks for men?   *For each question, collect three of four responses, and then move on.*   1. What do people in your community think about men who take part in transactional sex with girls?   *Let them express themselves freely, then probe around community judgements if necessary. For example, do people admire these men? Do they think that these men care about girls?*  *You can also ask:*   1. What do adults in your community think about men who take part in transactional sex? 2. What do teachers, community leaders, or other professionals think about men who take part in transactional sex?   *For each question, collect three of four responses, and then move on.*   1. What do people in your community think about men who do not take part in transactional sex with girls?   *Let them express themselves freely, then probe around community judgements if necessary. You can ask similar questions: do people admire these men? Do they think that these men care about girls?*  *You can also ask:*   1. What do adults in your community think about men who do not take part in transactional sex? 2. What do teachers, community leaders, or other professionals think about men who do not take part in transactional sex?   *For each question, collect three of four responses, and then move on.*   1. Do you think listening to the radio drama has changed at all the way you think about men taking part in transactional sex? |
| *10 mins: Responsibility to prevent transactional sex* |
| 1. What do you think could be done to support girls to avoid these risks?   *Let them express themselves freely, then if necessary probe around support at school, at home, by the government...?*  *Try to encourage discussion between participants about this. Aim to get a range of views from the participants.*   1. Who in the community can support them, and how?   *If they don’t mention a specific group, ask about peers, parents, teachers...?*  *For each question, collect three of four responses, and then move on.* |
| *5 mins: Closing the discussion* |
| We have come to the end of the discussion. Thank you for helping us understand more about your views. We have talked about many things today. Please remember that we agreed to keep this discussion confidential. Please do not share what was said here with others outside the group.  People will be curious and you may have to say something. I suggest you tell them that we were discussing how to improve a radio drama. Please do not give further details, so that we can maintain confidentiality.  How does that sound to you? Do you have questions for me? If you would like to speak with me further in private, I will be here for a time after we end. If you have questions on another day, you can contact the study supervisor Revocatus Sono.  Thank you for your time. |
